# Minutes of Ohio State Dental Society

**Published:** 1900-03-15

**Authors:** 


### MINUTES OF OHIO STATE DENTAL SOCIETY.

The Society met in the Great Southern Hotel at Columbus, Ohio, at 11 a. m., Tuesday, December 5, 1899. At this session no business was transacted.

The afternoon session was called at 2 p. m., at which time the president's address, by Dr. L. P. Bethel, was read and, on motion, referred to a committe for report.

Dr. G. S. Junkerman then read a paper entitled "A

Hydro-Mechanical Theory of Sensitive Dentin," which brought out an interesting discussion. (See pages 49 and 55.)

Dr. C. D. Peck reported for Dr. J. K. Douglas a case of third superior molar in the antrum. Other like cases were reported by Drs. Fletcher, Van Doorn and Cook.

Mr. Thos. A. Long, of Philadelphia, read an interesting paper on "Japanese Dental Art, Ancient and Modern," (See page 42, January number) accompanied with a large collection of specimens owned by the S. S. White Dental Manfg. Co.

The following officers were elected for the ensuing year: President, Dr. L. L. Barber; First Vice-President, Dr. H. F. Harvey; Second Vice-President, Dr. Otto Arnold; Secretary, Dr. S. D. Rugnles; Treasurer, Dr. C. I. Keely; Directors, for three years, Drs. J. R. Callahan, O. M. Heise, Henry Barnes and E. D. Scheble.

At the evening session Dr. W. A. Price gave a lecture and demonstration on "Roentgen Rays as Applied to Dentistry." (See page 84.)

The following new members were elected:

| | |
|---|---|
| R. A. Suhr, Cleveland, | J. F. Knowlton, Zanesville, |
| D. Stern, Cincinnati, | H. E. Jenkins, Ironton, |
| W. E. Wilkinson, Arcanum, | C. H. Lucas, Plain City, |
| S. M. Weaver, Cleveland, | G. H. Mengel, Bucyrus, |
| C. H. Birkett, E. Liverpool, | J. H. Riggle, Sherrodsville, |
| P. F. Schoff, Dayton, | W. H. Kepner, Bucyrus, |
| C. M. McElroy, Delaware, | M. L. Williamson, Marietta. |

Dr. H. J. Custer read a paper on "Empyema of the Maxillary Sinuses, Complicated by Empyema of Contiguous Sinuses," with presentation of cases. (See page 129, March number.)

Dr. M. H. Fletcher read a paper on "Alkaline Saliva." (See page 122, March number.)

Dr. Harvey reported and demonstrated a new form of clamp for retaining the rubber dam in place, made from piano wire. (See page 143, March number.)

The officers-elect were installed, and the session adjourned to 10 A. M. next day.

Dr. Taft, on behalf of the society, in fitting remarks presented Dr. J. B. Bauman a purse of $75 as a token of sympathy because of the accident at the clinic, one year ago, which cost him the use of his hand.

The committees for the ensuing year are as follows:

*Executive*—Drs. H. Barnes, H. T. Smith, Chas. Welch, L. P. Bethel.

*Arrangements*—Drs. W. H. Todd, Otto Arnold, A. F. Emminger.

*Clinics*—Drs. G. H. Wilson, H. C. Brown, E. D. Scheble, W. S. Locke.

*Publication*—Drs. L. P. Bethel, J. K. Smith, W. T. Born, W. T. McLean.

*Membership*—Drs. J. R. Callahan, Grant Molyneaux, O. N. Heise, L. T. Canfield.

*Dental Legislation*—Drs. A. F. Emminger, J. R. Callahan, M. H. Fletcher, O. N. Heise, Geo. H. Wilson.

*Army and Navy*—Dr. Otto Arnold.

*Hygiene for Institutions*—Drs. W. A. Price, J. Taft, W. H. Todd, L. P. Bethel, L. L. Barber.

The Board of Directors passed the following resolution:

WHEREAS, The annual revenue of the Society at present is in excess of its needs; therefore be it

*Resolved*, That the membership dues be reduced to three dollars, and the annual dues to two dollars.

The Directors appropriated $300 for legal purposes, to be used should occasion require it.

The Committee on Legislation was directed to confer with and enlighten the legislators from every county in the State on dental legislative measures which may be introduced into the Legislature.

Adjourned to December, 1900.

